# Safety of one 8.5-Fr pigtail catheter for postoperative continuous open gravity drainage after uniportal video-assisted thoracoscopic surgery pneumonectomy

**DOI:** 10.1186/s13019-024-02894-6

**Published:** 2024-07-18

**Authors:** Xiang-Long Kong, Yue- Zhang, Yu- Jia, Bo-Xiong Ni, Mingyu- Wang, Xiang-Yuan Jin, Hai Xu, Shi-Dong Xu

**Affiliations:** 1 Department of Thoracic Surgery, Harbin Medical University Cancer Hospital, No. 150, Hapin Road, Harbin, 150081 China; 2 Department of Area B, ICU, The Second Affiliated Hospital of Harbin Medical University, Harbin, China

**Keywords:** Chest tube, Uniportal video-assisted thoracoscopic surgery, Pneumonectomy, Postoperative drainage

## Abstract

**Objectives:**

Uniportal video-assisted thoracoscopic surgery pneumonectomy (U-VATS-P) is feasible and safe from a perioperative standpoint. How to choose the proper chest tube and drainage method is important in enhanced recovery after surgery (ERAS) protocols. In this study, we aimed to assess the safety of one 8.5-Fr (1Fr = 0.333 mm) pigtail catheter for postoperative continuous open gravity drainage after U-VATS-P.

**Methods:**

We retrospectively reviewed a single surgeon’s experience with U-VATS-P for lung cancer from May 2016 to September 2022. Patients were managed with one 8.5-Fr pigtail catheter for postoperative continuous open gravity drainage after U-VATS-P. The clinical characteristics and perioperative outcomes of the patients were retrospectively analyzed.

**Results:**

In total, 77 patients had one 8.5-Fr pigtail catheter placed for postoperative continuous open gravity drainage after U-VATS-P for lung cancer. The mean age was 60.9$\pm$7.39 (40–76) years; The mean FEV1 was 2.1$\pm$0.6 (l/s), and the mean FEV1% was 71.2$\pm$22.7. The median operative time was 191.38$\pm$59.32 min; the mean operative hemorrhage was 109.46$\pm$96.56 ml; the mean duration of postoperative chest tube drainage was 6.80$\pm$2.33 days; the mean drainage volumes in the first three days after operation were 186.31$\pm$50.97, 321.97$\pm$52.03, and 216.44$\pm$35.67 ml, respectively; and the mean postoperative hospital stay was 7.90$\pm$2.58 days. No patient experienced complications resulting from chest tube malfunction. Ten patients experienced minor complications. One patient with nonlife-threatening empyema and bronchopleural fistula required short rehospitalization for anti-inflammatory therapy and reintubation. Three patients with chylothorax were treated with intravenous nutrition. Four patients had atrial fibrillation that was controlled by antiarrhythmic therapy. Two patients had more thoracic hemorrhagic exudation after the operation, which was found in time and was cured effectively, so they were discharged from the hospital uneventfully after early hemostatic therapy and nutritional support.

**Conclusions:**

All patients in this study received early postoperative rehabilitation, and the rate of relevant complications was low. We therefore recommend a single 8.5-Fr pigtail catheter for postoperative continuous open gravity drainage as an effective, safe and reliable drainage method for the management of U-VATS-P.

## Introduction

Pneumonectomy performed by uniportal video-assisted thoracoscopic surgery does not compromise perioperative mortality or long-term outcomes in clinical practice [1, 2], but each thoracic surgery department implements its own protocol for the management of pneumonectomy, and few reports have described the use of small-hole drainage or effective drainage methods for pneumonectomy. Traditionally, a large-bore chest tube, such as a 28-Fr or 32-Fr tube, is inserted into the chest wall at the proper depth after pneumonectomy (usually 4–5 cm) and clipped and opened intermittently according to the position of the trachea and mediastinum in order to maintain the pressure balance of the thorax [3, 4]. Recently, small-hole drainage has been increasingly considered owing to the extensive use of VATS and ERAS [5, 6]. We have therefore modified our chest tube management protocol from traditional drainage to a protocol combining one 8.5-Fr pigtail catheter with continuous open gravity drainage, starting in May 2016. Some studies have reported the successful application of pigtail drainage in the treatment of VATS lobectomy and continuous open drainage after pneumonectomy, but none has evaluated the safety of small-caliber drainage catheters for continuous open gravity drainage after U-VATS-P [7–9]. In this study, we aimed to assess the safety of one 8.5-Fr pigtail catheter for postoperative continuous open gravity drainage after U-VATS-P.

## Methods

The medical data of 77 patients with lung cancer who underwent U-VATS-P and systematic mediastinal lymph node dissection and were managed with one 8.5-Fr pigtail catheter for postoperative continuous open gravity drainage in the Department of Thoracic Surgery, Harbin Medical University Cancer Hospital, between May 2016 and September 2022 were retrospectively analyzed. All the operations were performed by the same thoracic surgical team. This study was approved by the institutional review board of Harbin Medical University Cancer Hospital. As this was a retrospective analysis, written informed consent from each patient was not needed.

**Fig. 1:**
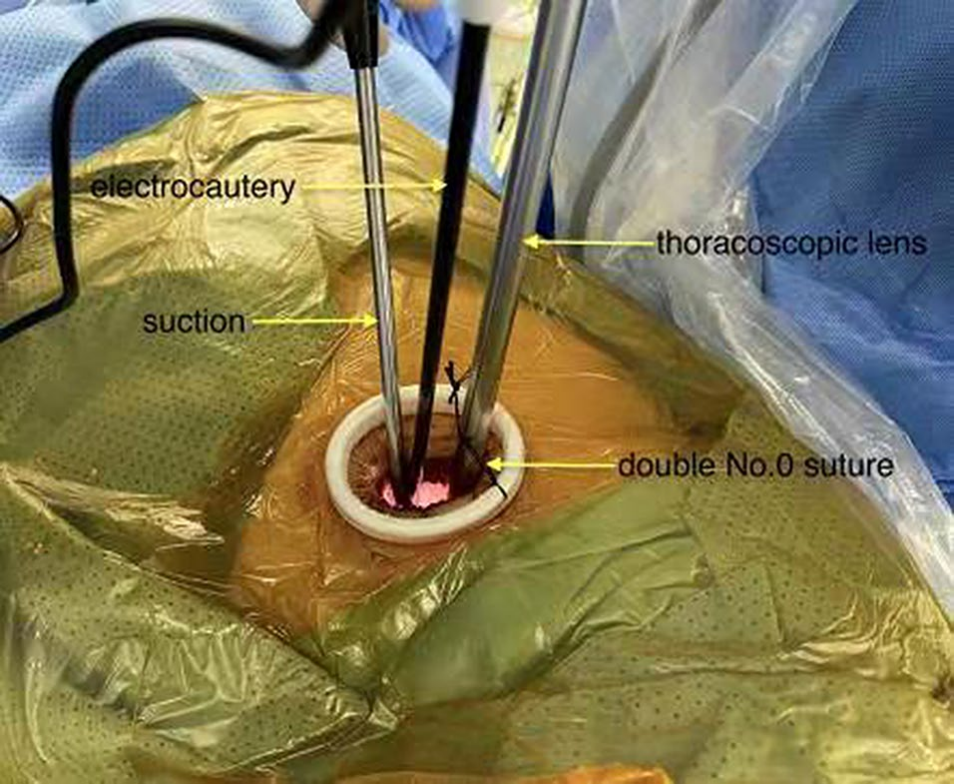
An incision about 4.0 cm in length was made at the 5th intercostal space between the anterior axillary line and middle axillary line. It was protected with a wound retractor

All of the patients received general anesthesia by intravenous induction. The patients were placed in the lateral decubitus position and then intubated with a double-lumen endotracheal tube or single-lumen endotracheal tube combined with bronchial occluder to perform single-lung ventilation. After disinfection, an incision approximately 4.0 cm in length was made at the 5th intercostal space between the anterior axillary line and middle axillary line and protected with a wound retractor (Fig. 1). The assistant stood on the opposite side of the operator and held the thoracoscopic lens, which was limited by the double No. 0 suture. The left incision was done backward to avoid interference of the visual field by the heart. The procedure was performed without rib spreading, and the vein and artery were divided anatomically using the electrocoagulation hook and dissected separately using endostaplers from different angles through the single incision. The small space limited the placement angles of the endostaplers, so the cooperation of assistants and the reasonable placement of surgical instruments were crucial. The principal bronchus was fully exposed and transected using the endostapler by pulling it to a proper angle. We found that because there was not enough space between the aortic arch and the left main bronchus, it was difficult to disconnect the left principal bronchus with a 4.0-cm incision. We had to fully dissociate the left principal bronchus in advance and pull it up forcefully to find a proper angle between the endostapler and the bronchus, and finally clamp and disconnect the bronchus.

**Fig. 2:**
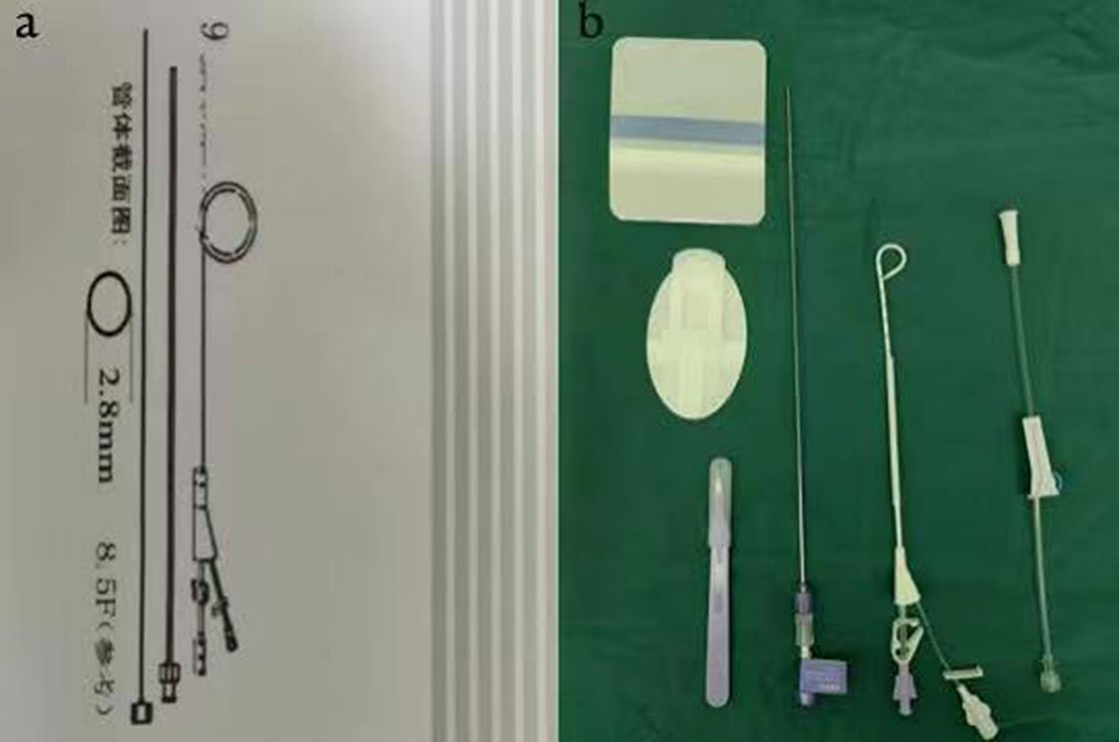
The appearance of the 8.5-Fr pigtail catheter. **a** Schematic diagram showing the 8.5-Fr pigtail catheter. **b** Display of various components of the 8.5-Fr pigtail catheter

**Fig. 3:**
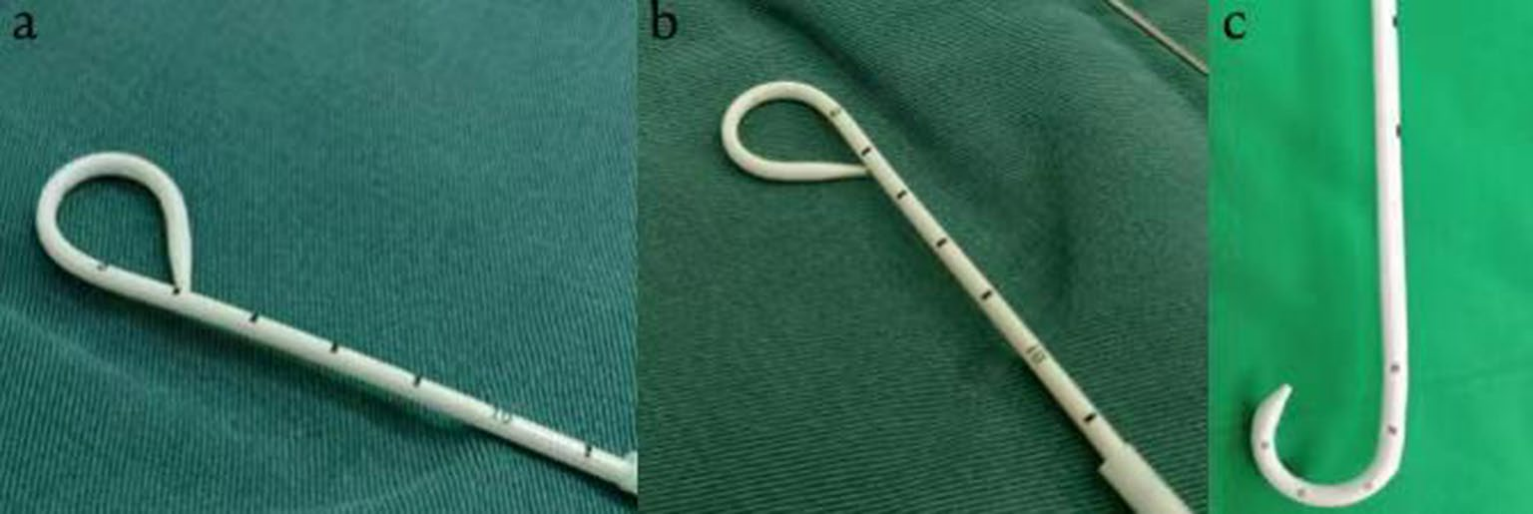
Multiple drainage holes are on the inner surface of the pigtail ring rather than on the side, so tube plugging rarely occurs. **a b** Display of the inner side of the 8.5-Fr pigtail catheter. **c** Display of the side of another kind of pigtail ring

**Fig. 4:**
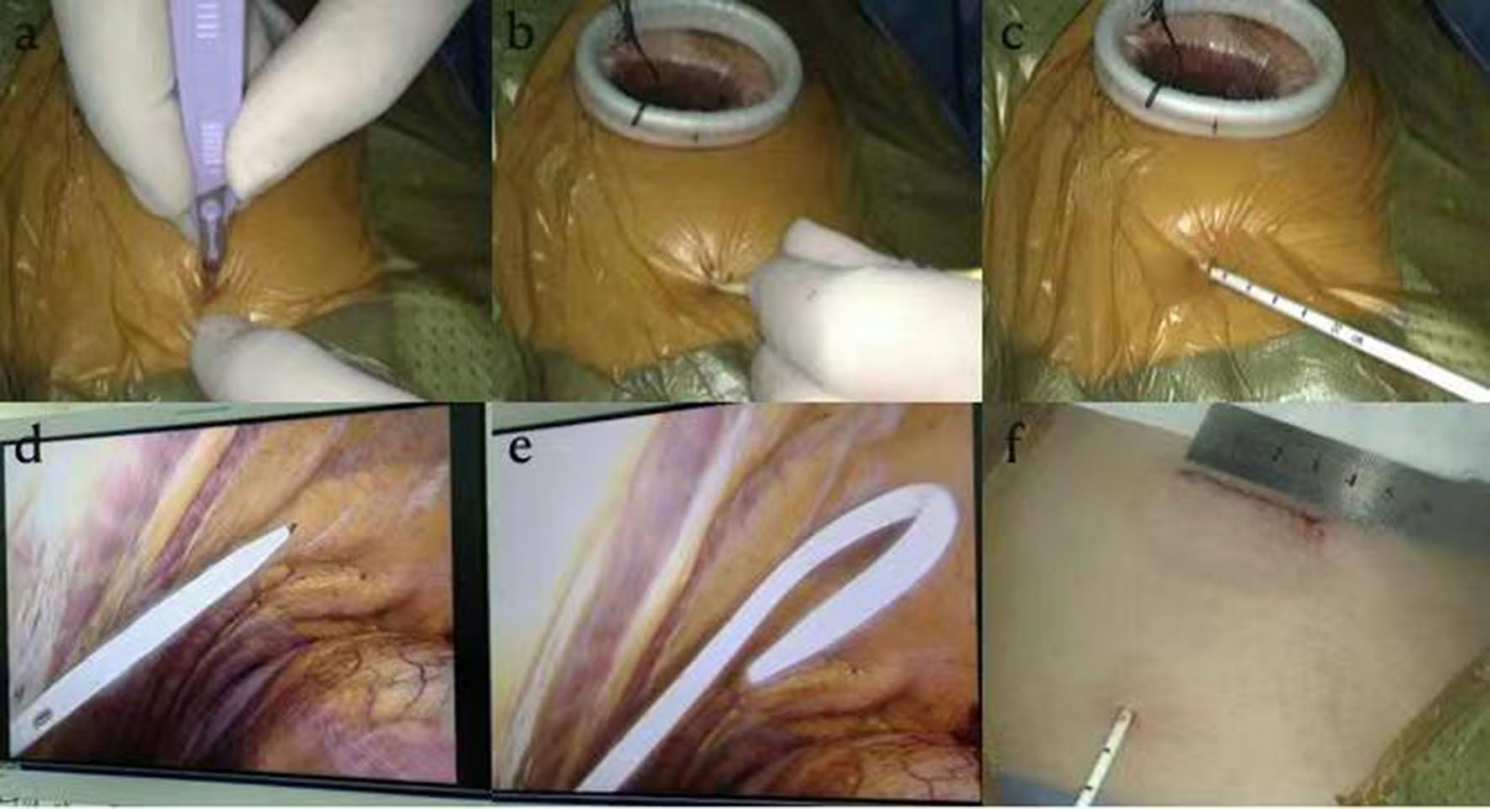
The procedure to perform the insertion of the 8.5-Fr pigtail catheter. **a** Use a sharp blade to cut a 2-mm incision on the skin of the 7th intercostal space of the posterior axillary line. **b c d** Putting the 8.5-Fr pigtail catheter into the chest cavity upward and backward together with the guiding device. **e** After the guide device is removed, the tip of the catheter is shaped like a pigtail ring. **f** The depth of insertion into the thoracic cavity is 15 cm

**Fig. 5:**
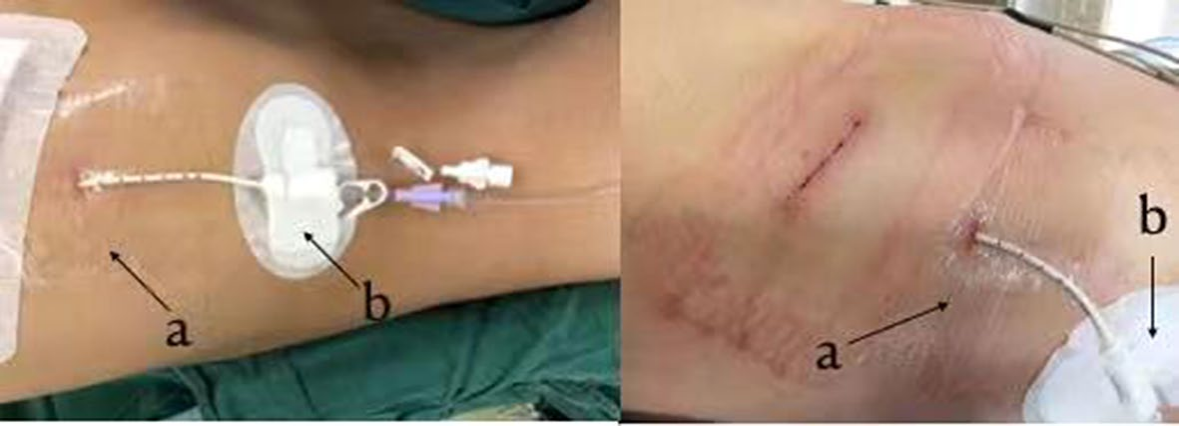
Fixation of the 8.5-Fr pigtail catheter on the skin. **a** Transparent adhesive dressing membrane. **b** Special drainage tube–fixing sticker

Finally, we inspected for bronchopleural fistula by using the water seal test intraoperatively. At the end of the operation, a single 8.5-Fr pigtail catheter (connected with a water-sealed drainage bottle, without external suction and intermittent clamping) was placed in the 7th intercostal space of the posterior axillary line in each patient (Fig. 2). The wall of the 8.5-Fr pigtail catheter was smooth, with strong anticoagulation ability and good flexibility. Multiple drainage holes were located on the inner surface of the pigtail ring rather than on the side to prevent poor drainage (Fig. 3). The procedure to perform the insertion of the 8.5-Fr pigtail catheter was simple and was divided into the following steps (Fig. 4): (I) We used a sharp blade to cut a 2-mm incision into the skin of the 7th intercostal space of the posterior axillary line. (II) We put the 8.5-Fr pigtail catheter into the chest cavity upward and backward together with the guiding device, the tip of which was designed as a blunt head to avoid damaging the intercostal artery. (III) The depth of insertion into the thoracic cavity was 15 cm. After the guide device was removed, the tip of the catheter was shaped like a pigtail ring. After the operation, we fixed the 8.5-Fr pigtail catheter on the skin with a transparent adhesive dressing membrane and special drainage tube–fixing sticker instead of a suture (Fig. 5).

After operation, the patient returned to the regular exam room for electrocardiographic monitoring, analgesia, and application of a 1,500 ml intravenous fluid restriction and metoprolol, digoxin and furosemide to avoid acute postpneumonectomy pulmonary edema [10]. If the patient had obvious postoperative chest pain, 30–60 mg nonsteroidal anti-inflammatory drug (ketorolac) was injected intramuscularly every 4 to 6 h, according to the patient’s age and weight during the first two days after surgery. From the third day after surgery on, we switched to oral nonopioid analgesics (three times/day) and adjusted the drug dosage and administration time according to the specific situation. No additional analgesics were administered before or after chest tube removal because patients do not feel pain during the extubation process.

**Fig. 6:**
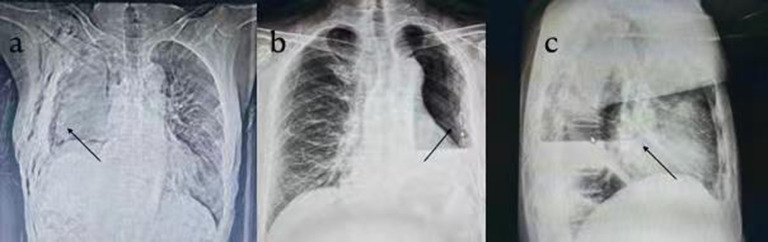
Chest radiography performed on Day 2 after U-VATS-P in patients who received one 8.5-Fr pigtail catheter for postoperative continuous open gravity drainage. The arrow indicates that one 8.5-Fr pigtail catheter was placed in the thoracic cavity. **a** The patient underwent right U-VATS-P. **b c** The patient underwent left U-VATS-P

**Fig. 7:**
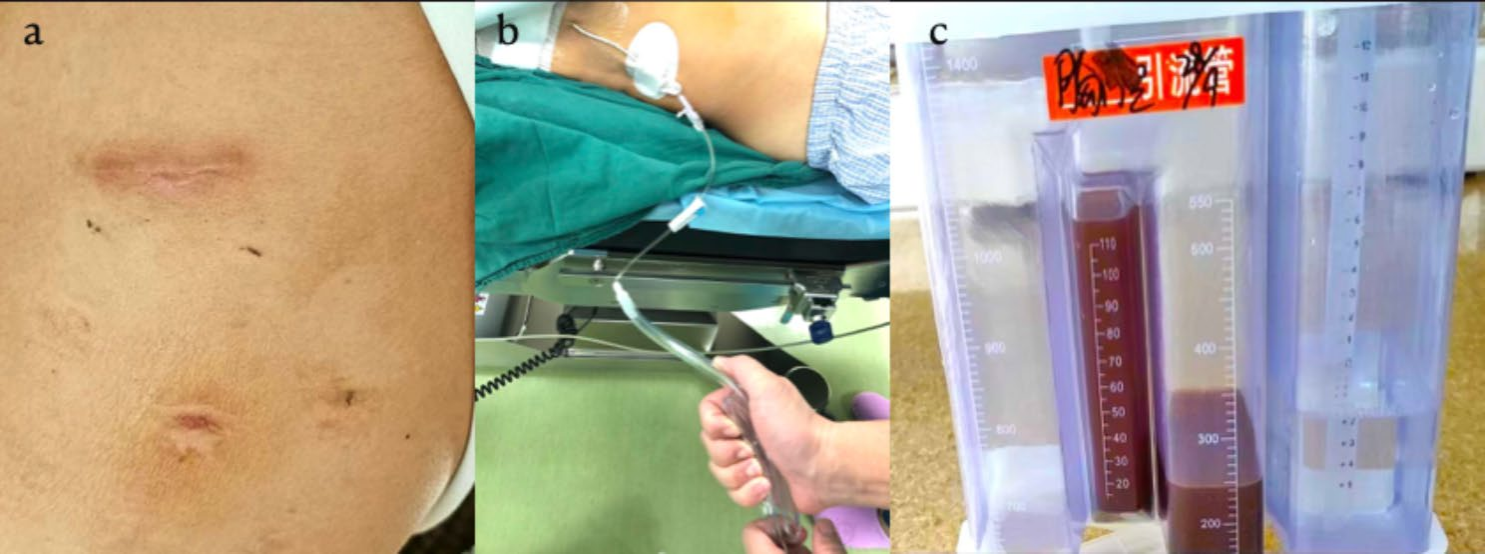
**a** The incision and drainage nozzle healed well. **b** The nurse squeezed the thick chest tube connecting the chest bottle to keep the 8.5-Fr pigtail catheter unobstructed by taking advantage of the sudden change in pressure in the chest tube. **c** The fluctuation range of the water column in the drainage bottle was small, within 4 cm of the water column, even when the patient coughed hard

All patients underwent chest X-ray on the second day after operation (Fig. 6). Nursing care of thoracic drainage was provided to ensure nonobstruction of the pigtail catheter. Indications for chest tube removal included a volume of drainage drop below 100 ml in 24 h, chest X-ray showing that the pleural effusion had faded below the pulmonary hilar and showing the absence of intrathoracic hemorrhage and air leakage. Extubation of the 8.5-Fr pigtail catheter remained more convenient and did not require the patient to hold his breath. After extubation, the incision was closed naturally, and the postoperative scar was very small, almost invisible (Fig. 7a). The patient was discharged after achieving a stable condition without needing further treatment.

The observation indicators included general data, lung function, blood gas analysis operation time, operative hemorrhage, daily postoperative drainage volume in the first three days, drainage days, postoperative hospital stay, and postoperative complications (including bronchopleural fistula, chylothorax, cardiac arrhythmia, chest tube reinsertion, and intrathoracic hemorrhage). SPSS 20.0 software was used to statistically analyze the data. Continuous variables are expressed as mean ± standard deviation (SD).

## Results

From May 2016 to September 2022, a total of 77 patients underwent placement of one 8.5-Fr pigtail catheter for postoperative continuous open gravity drainage after U-VATS-P for lung cancer. The clinical characteristics and perioperative outcomes of the patients and their tumors are listed in Table 1. There were 60 male patients and 17 female patients. We chose forced expiratory volume in 1 s (FEV1) and FEV1% to assess pulmonary function, as these are by far the most frequently used parameters for assessing respiratory function in our specialty. The mean FEV1 was 2.1$\pm$0.6 (l/s), and the mean FEV1% was 71.2$\pm$22.7. The blood gas levels of all patients were within the normal ranges, showing no electrolyte disorders, carbon dioxide retention or hypoxemia. Three patients received preoperative neoadjuvant therapy, and three patients had extensive dense pleural adhesion during operation. Surgical margins were negative in all patients. The mean age was 60.9$\pm$7.39 (40–76) years old; the mean operative time was 191.38$\pm$59.32 min; the mean operative hemorrhage was 109.46$\pm$96.56 ml; the mean duration of postoperative chest tube drainage was 6.80$\pm$2.33 days; and the mean postoperative hospital stay was 7.90$\pm$2.58 days. The use of one 8.5-Fr pigtail catheter to drain the empty hemithorax following U-VATS-P was found to drain well and maintain the pressure balance of the thorax. The mean drainage volumes in the first three days after operation were 186.31$\pm$50.97, 321.97$\pm$52.03, and 216.44$\pm$35.67 ml, respectively. No patient experienced complications resulting from chest tube malfunction. Ten patients experienced minor complications. One patient with non-life-threatening empyema and bronchopleural fistula required short rehospitalization for anti-inflammatory therapy and reintubation. Three patients with chylothorax were treated with intravenous nutrition. Four patients had atrial fibrillation that was controlled by antiarrhythmic therapy. Two patients had more thoracic hemorrhagic exudation after operation that could be found in time and was cured effectively, so they were discharged from the hospital uneventfully after early hemostatic therapy and nutritional support. No poor wound healing occurred.

**Table 1:** Clinical characteristics and perioperative outcomes of patients and tumors

Characteristics	U-VATS-P for lung cancer
**Age**	
Mean (range)	60.9$\pm$7.39 (40–76 years)
**Sex**	
Male	60
Female	17
Tumor location	
Left	61
Right	16
**Lung function**	
FEV1 (l/s)	2.1$\pm$0.6
FEV1%	71.2$\pm$22.7
Neoadjuvant therapy	3
**Adhesion**	
Slight	1
Severe	2
**Accumulative thoracic drainage (ml)**	
POD 1	186.31$\pm$50.97
POD 2	321.97$\pm$52.03
POD 3	216.44$\pm$35.67
Average operative duration (minutes)	191.38$\pm$59.32
Operative hemorrhage (ml)	109.46$\pm$96.56
Duration of postoperative chest tube drainage (days)	6.80$\pm$2.33
Postoperative hospital stay (days)	7.90$\pm$2.58
**Pathological types**	
Squamous cell carcinoma	49
Adenocarcinoma	18
Other	9
**Complications**	
Bronchopleural fistula	1
Chylothorax	3
Cardiac arrhythmia	4
Active thoracic hemorrhage	0
Postoperative thoracic hemorrhagic exudation	2
Chest tube reinsertion	1
Poor wound healing	0

Continuous variables are expressed as mean ± standard deviation (SD)

POD postoperative days

FEV1: forced expiratory volume within the first second

In our clinical practice, we have observed that there are indeed differences in the postoperative recovery process between patients undergoing left pneumonectomy and right pneumonectomy. Although the surgical procedures for both are the same, the risk of right pneumonectomy is often higher, and the incidence of postoperative mediastinal oscillation and bronchopleural fistula is also higher. Therefore, we compared the drainage volume, drainage time, length of hospital stay, and postoperative complications of patients undergoing left vs. right U-VATS-P in this article. The results are listed in Table 2. Through this comparison, we found no significant difference between the two, which may be due to the small sample size and our screening parameters: After all, patients undergoing U-VATS-P usually have relatively good chest anatomical conditions.

**Table 2:** Comparison of perioperative outcomes of patients undergoing left or right U-VATS-P

Variables	Left U-VATS-P (61 patients)	Right U-VATS-P (16 patients)	P value
Total drainage volume (ml)	1472.23$\pm$692.75	1607.87$\pm$619.43	> 0.05
Drainage time (days)	6.25$\pm$2.96	7.0$\pm$2.79	> 0.05
Hospital stay (days)	7.46$\pm$2.59	8.$52\pm$2.71	> 0.05
Postoperative complications	8	3	> 0.05

Continuous variables are expressed as mean ± standard deviation (SD) unless otherwise indicated.

## Discussion

For central lung cancer, which often involves the hilum, pneumonectomy is one of the means of treatment that has the opportunity to cure the disease, which can substantially prolong the survival time of patients [1, 11]. However, due to the high trauma of the operation, pneumonectomy is often (20–60%) followed by postoperative complications, such as low-volume circulatory disorder, pulmonary edema, pneumonia, and acute respiratory failure [2, 3, 12]. Eric Yu Wei Lo et al. concluded that compared with clamp-release drainage, balanced chest drainage results in a lower incidence of postpneumonectomy pulmonary edema and death [3]. This implies that the management of drainage tubes after pneumonectomy is very important. There is an urgent need for a simple and effective drainage method to reduce the incidence of postoperative complications after pneumonectomy. In recent years, as thoracoscopic surgery technology and the concept of ERAS have advanced, approaching pneumonectomy by minimally invasive approaches has not negatively impacted perioperative outcomes, and the incidence of complications such as empyema, mediastinal shift and major bleeding requiring reopening has decreased [12]. There is no consensus on the management of pneumonectomy postoperative drainage. Each thoracic surgery department implements its own protocol for the management of pneumonectomy postoperative drainage in clinical work. Some thoracic surgeons tend to use small-bore chest tubes after pneumonectomy, which allow for good drainage and fewer complications, and modify the chest tube management protocol from intermittent chest tube clamping to continuous open gravity drainage or even a no-drainage system [6, 13–16].

Most of the previous findings for small-bore chest tubes are applicable to patients undergoing VATS lobectomy or segmentectomy, but data for patients undergoing VATS pneumonectomy are limited [17–19]. Yongbin Song et al. concluded that compared with a 24-Fr chest drainage tube, the application of an 8-Fr ultrafine chest drainage tube after thoracoscopic lobectomy significantly shortened the drainage time, reduced the total drainage volume, reduced the postoperative pain degree, shortened the hospital stay, and effectively detected postoperative intrathoracic hemorrhage [8].

Although small-bore chest tubes have obvious advantages [20, 21], many people still have doubts about their application in patients undergoing pneumonectomy, such as whether the chest tube is easy to block, whether the drainage effect is good, whether it can be detected immediately when active chest bleeding occurs, and whether complications such as mediastinal shift and pulmonary edema will become more common after surgery. Our center started using a single 8.5-Fr pigtail catheter for postoperative continuous open gravity drainage after U-VATS-P in May 2016 and achieved a good drainage effect. The inner wall of the 8.5-Fr pigtail catheter has a strong anticoagulant coating and multiple drainage holes on the inner surface of the pigtail ring rather than on the side, so tube plugging rarely occurs. Every morning after the operation, the nurse squeezed the thick chest tube connecting the chest bottle to keep the 8.5-Fr pigtail catheter unobstructed by using the sudden change in pressure in the chest tube (Fig. 7b).

In this study, we retrospectively analyzed the data of 77 cases in which we used one 8.5-Fr pigtail catheter for postoperative continuous open gravity drainage after U-VATS-P. All patients received early postoperative rehabilitation, and the rate of relevant complications was low. The mean drainage volumes in the first three days after the operation were 186.31$\pm$50.97, 321.97$\pm$52.03, and 216.44$\pm$35.67 ml, respectively, which indicated that the 8.5-Fr pigtail catheter was very effective. Two patients had more thoracic hemorrhagic exudation after operation that could be found in time and were cured effectively after early hemostatic therapy and nutritional support, with no need for a second surgery. Three patients with chylothorax improved after receiving intravenous nutrition treatment and were extubated. During the treatment, an average total drainage volume of 2600 ml pleural effusion was drawn out through the 8.5-Fr pigtail catheter. From our clinical experience, this drainage effect is similar to that of the traditional thick chest tube [13]. In the past three years of clinical work, our center has routinely used an 8.5-Fr pigtail catheter in VATS lobectomy and conventional thoracotomy pneumonectomy, and the drainage effect has been very good.

In this study, no patient experienced life-threatening complications, such as mediastinal shift and pulmonary edema. The fluctuation range of the water column in the drainage bottle was small, within 4 cm of the water column, even when the patient coughed hard (Fig. 7c). Therefore, the 8.5-Fr pigtail catheter allows the empty hemithorax to maintain stable pressure and prevent mediastinal shifting. Because of the double fixation of the transparent adhesive dressing membrane and special drainage tube fixing sticker, no drainage tube fell off. The postoperative pain response of all patients in this study was mild, and the incision and drainage nozzle healed well.

## Limitations

This study has several limitations. It was a retrospective single-institute study in a small study population. Finally, in this study, there were no statistical data on patients who were converted to thoracotomy due to severe thoracic adhesion, large tumors or severe pulmonary conus invasion. Therefore, the statistical analysis was not robust, and the safety of a single 8.5-Fr pigtail catheter for postoperative drainage after U-VATS-P reported in this study needs to be confirmed in a prospective large-scale multicenter study.

## Conclusions

In conclusion, all the patients in this study received early postoperative rehabilitation, and the rate of relevant complications was low. Owing to the advantages of easy and safe intraoperative insertion and postoperative removal of the 8.5-Fr pigtail catheter, as well as continuous open postoperative drainage without the need for intermittent clamping, the excellent drainage effect, and the good drainage healing, we recommend one 8.5-Fr pigtail catheter for postoperative continuous open gravity drainage as an effective, safe and reliable drainage method for the management of U-VATS-P.

## Data Availability

The data and materials used in the current study are available from the corresponding author upon reasonable request.

## Abbreviations

VATS: Video assisted thoracoscopic surgery
U-VATS-P: Uniportal video-assisted thoracoscopic surgery pneumonectomy
ERAS: Enhanced recovery after surgery
POD: Postoperative days
SD: Standard deviation
